# The Principles on Which the Treatment of Cholera Should Be Based

**Published:** 1853

**Authors:** 


*The Principles on which the Treatment of Cholera should be based.*

Dr. Snow read a paper on this subject before the Medical Society of London, January 21, 1854. He said that the absence of settled opinions respecting the nature of cholera was the cause of the various and contrary plans on which it was treated. In the greater number of epidemic or self-propagating diseases, the morbid poison entered the blood in some way, and after multiplying itself during a period of so-called incubation, it affected the whole system, the illness commencing by fever and other general symptoms. Cholera, on the other hand, commenced with an effusion of fluid into the alimentary canal, without any previous illness whatever, and the subsequent symptoms were the result of the change in the blood occasioned by this effusion of its watery part. The analyses of the blood of the chelera patients, performed by Dr. O'Shaughnessy, Dr. Garrod, and others, proved that its thick and tarry condition was caused by the loss of a great part of its water, together with a portion of its saline constituents. The physical state of the blood prevented it from passing through the capilliaries of the lungs, except in very small quantity, and these occasioned the symptoms of asphyxia; whilst the arteries throughout the body, being almost deprived of blood from the same cause, produced the coldness and other symptoms of collapse. These circumstances indicated that the immediate action of the cholera poison was confined to the alimentary canal, and this view was confirmed by the circumstance that all the general symptoms could be removed for a time by the injection of a weak saline solution into the veins, which merely replaced the portion of the blood which had been lost, and could not remove the effects of a poison circulating in that fluid. The preliminary diarrhœa with which the greater number of cholera cases commenced, could generally be cured by the ordinary remedies for diarrhœa, which could not have any effect on a poison circulating in the blood. The evidence respecting the mode of communication of cholera that he had brought before the society on a former occasion tended also to show that the materies morbi entered the alimentary canal by being accidentally swallowed, and there propagated itself, and was discharged in the evacuations. The following were the principles of treatment which the above view of the pathology of cholera suggested:

1. Medicines should be chosen which have the effect of destroying low forms of organized beings, and of preventing fermentation, putrefaction, and other kinds of molecular change in organic matter. Prepared animal charcoal, sulphur, and creosote were amongst the agencies which deserve a more extended trial.

2. The remedies should be administered with a view to their action in the stomach and bowels, and not to their being absorbed.

3. They should be given in such quantities and in such a form as to insure, as much as possible, their application to the whole surface of the alimentary tube.

4. These medicines should be continued till there was no danger of a return of the purging.

5. It was useless and injurious to attempt to bring the patient out of the state of collapse by stimulants and the application of heat, and they should give watery drinks, and be content to wait till they were absorbed, unless in desperate cases, in which it might be desirable to inject into the bloodvessels a weak saline solution, resembling the portion of the blood which had been lost.

Lancet, January 28, 1854.

